# Space Radiation Alters Genotype–Phenotype Correlations in Fear Learning and Memory Tests

**DOI:** 10.3389/fgene.2018.00404

**Published:** 2018-10-09

**Authors:** Ovidiu Dan Iancu, Sydney Weber Boutros, Reid H. J. Olsen, Matthew J. Davis, Blair Stewart, Massarra Eiwaz, Tessa Marzulla, John Belknap, Christina M. Fallgren, Elijah F. Edmondson, Michael M. Weil, Jacob Raber

**Affiliations:** ^1^Department of Behavioral Neuroscience, Oregon Health & Science University, Portland, OR, United States; ^2^Department of Environmental and Radiological Health Sciences, Colorado State University, Fort Collins, CO, United States; ^3^Departments of Neurology and Radiation Medicine, Division of Neuroscience, ONPRC, Oregon Health & Science University, Portland, OR, United States

**Keywords:** space radiation, genotype–phenotype, behavioral genetics, fear learning and memory, mice

## Abstract

Behavioral and cognitive traits have a genetic component even though contributions from individual genes and genomic loci are in many cases modest. Changes in the environment can alter genotype–phenotype relationships. Space travel, which includes exposure to ionizing radiation, constitutes environmental challenges and is expected to induce not only dramatic behavioral and cognitive changes but also has the potential to induce physical DNA damage. In this study, we utilized a genetically heterogeneous mouse model, dense genotype data, and shifting environmental challenges, including ionizing radiation exposure, to explore and quantify the size and stability of the genetic component of fear learning and memory-related measures. Exposure to ionizing radiation and other external stressors altered the genotype–phenotype correlations, although different behavioral and cognitive measures were affected to different extents. Utilizing an integrative genomic approach, we identified pathways and functional ontology categories associated with these behavioral and cognitive measures.

## Introduction

A unique feature of the space radiation environment is the presence of galactic cosmic rays (GCRs) and solar particle events (SPEs). GCR involves protons and fully ionized atomic nuclei such as ^56^Fe, while SPE includes predominantly low-to-medium energy protons with a small heavy ion component. These exposures pose a significant hazard to space flight crews not only during the mission but also at later times after the mission when slow-developing adverse effects could finally become apparent. The hazards associated with the space environment will likely impact many organs, including the brain.

In people exposed to irradiation, the nature and extent of behavioral and cognitive changes is variable and cannot easily be predicted on the basis of radiation dose and type. This suggests the involvement of genetic factors. Conditions such as early-onset Alzheimer’s disease show simple Mendelian patterns of inheritance for which mutations in a single gene are necessary and sufficient to cause the disorder. In contrast, complex behavioral traits and their potential alteration by space irradiation are likely influenced by multiple genes. To date, radiation studies typically involved inbred strains of mice and rats, crosses of inbred mice to generate F1 mice, stocks with very limited genetic heterogeneity, or outbred mice or rats but without analysis of the genetic factors of the individual animals. The advent of advanced heterogeneous stocks, inexpensive high-throughput single-nucleotide polymorphism (SNP) genotyping, and new analytical approaches enables studying gene–environment interactions and genotype–phenotype relationships involved in radiation effects on the brain. Analyzing CNS radiation effects in genetically heterogeneous mouse populations therefore likely offer a better model for genetically diverse human populations. In this study, we utilized animals from the HS/Npt colony, which was developed in 1991 as a tool to investigate complex genetic traits and captures a significant amount of the genetic diversity that is available in *Mus musculus* (Roberts et al., 2007). These mice are now at G_70_, leading to further expansion of the genetic map.

Based on practical considerations of performing radiation studies at Brookhaven National Laboratories (BNLs) and the number of mice required for studying genotype–phenotype relationships, we searched for cognitive tests that: (1) allow testing relatively large groups of mice; (2) can be performed by mice of various genetic backgrounds, including HS/Npt mice; (3) are sensitive to detect effects of irradiation on the brain; and (4) are relevant with regard to environmental psychological and physical stressors astronauts experience during space missions. Contextual and cued fear learning and memory fit these criteria. They allow testing of relatively large groups of mice and can be used to distinguish hippocampus-dependent function (i.e., contextual fear) from hippocampus-independent, amygdala-dependent functions (i.e., cued fear) (Anagnostaras et al., 2001, 2010). Fear learning and memory has been assessed in mice of various genetic backgrounds (Paylor et al., 1994; Crawley et al., 1997; Owen et al., 1997; Wehner et al., 1997), including HS/Npt mice (Valdar et al., 2006). Fear learning and memory is also being used as cognitive test in humans (Milad et al., 2011) and is associated with trait vulnerability to anxiety (Indovina et al., 2011). Including controlled environmental emotional stressors in assessments of effects of space irradiation on brain function is relevant, as the environmental conditions astronauts experience during space missions include not only ionizing radiation but also psychological and physical stressors (Stutser, 2016). Exposure to space radiation might modulate the response to such stressors and this response might depend on the genetic background. Previous studies have shown that fear conditioning is sensitive to effects of gamma rays (Saxe et al., 2006; Olsen et al., 2014; Kugelman et al., 2016), ^28^Si ion irradiation (Raber et al., 2014, 2015b) and ^56^Fe ion irradiation (Villasana et al., 2010; Raber et al., 2013) on hippocampus-dependent contextual fear memory and effects of gamma rays (Olsen et al., 2014), ^16^O ion irradiation (Raber et al., 2015a) on hippocampus-independent and amygdala-dependent cued fear memory, and effects of ^40^Ca ion exposure on locomotor baseline activity (prior to the first tone) and responses to tone and shock without affecting fear learning and memory (Raber et al., 2016). In this study, the effects of HZE ion or gamma ray irradiation on contextual and cued fear learning and memory were assessed in the genetically heterogeneous HS/Npt population, by irradiating mice with 0.4°Gy of 240 MeV/n ^28^Si or 600 MeV/n ^56^Fe ions or 3°Gy of ^137^Cs gamma rays, or sham irradiating them.

There are three possibilities arising from cognitive tests following ionizing radiation. Cognitive measurements could be strongly altered resulting in significant group mean differences. Second, cognitive measurements could be affected in more subtle ways mitigated by internal mechanisms. Third, ionizing radiation could have minimal effects on cognition. In the case of compensatory mechanisms, genetic factors are a distinct possibility. Genome-wide association studies have detected numerous genetic variants associated with complex behavioral and cognitive traits. For the most part, the QTL identified so far explain only a small proportion of phenotypic variability (Baud and Flint, 2017). Dissection of genetic architecture of complex traits such as behavioral and cognitive phenotypes has typically pursued the identification of individual Quantitative Trait Loci (QTLs); this approach has the great appeal and promise of identifying Quantitative Trait Genes (QTGs). However, in spite of the increased resolution of advanced crosses such as the HS/Npt, identification of QTGs remains an elusive goal since, to date, most QTLs contain multiple genes and in many cases hundreds of genes. In summary, traditional QTL analysis often identifies numerous QTL regions with small-to-moderate effect size and each genomic location can contain numerous genes although the utilization of advanced heterogeneous stocks such as the HS/Npt partially mitigates this situation.

Given these observations, our strategy for analyzing the HS/Npt-derived genetic, behavioral and cognitive data was twofold. First, we employed a distance/similarity based multi-locus technique that offers high flexibility in the number of loci included in the analysis (Excoffier et al., 1992; Wessel and Schork, 2006); this is further enhanced by machine learning techniques. Second, we focused on identifying biological pathways overrepresented in genes within significant loci by incorporating genomic annotation in the form of gene chromosomal locations and participation in functional groups.

## Materials and Methods

### Animals

HS/Npt is a reverse engineered outbred mouse population derived from the A/J, AKR/J, BALB/cJ, C3H/HeJ, C57BL/6J, CBA/J, DBA/2J, and LP/J strains as progenitors. The breeding colony consist of 48 families that are maintained by a circular breeding scheme that maximizes genetic heterogeneity. Three HS/Npt breeder mice of each family at generation 71, one male and two females, were provided by Dr. Robert Hitzemann, OHSU, and shipped to Colorado State University for breeding the experimental mice of the current study. Because of the limited number of breeders, matings were set up and litter generation was performed as follows. A first mating was set up which generated about 600 pups (cohort 1). When these animals reached 8–12 weeks of age they were shipped to BNL, Upton, NY, United States for irradiation. A second mating was then set up which generated about 1,200 pups (cohort 2) that were also shipped to BNL and similarly irradiated. Consequently, all of the mice in cohort 1 were from first litters, and those from cohort 2 were from second or third litters or first surviving litters from dam that had not previously had a surviving litter. Irradiation was either sham-irradiation or 0.4°Gy ^56^Fe (600 MeV/n), or 0.4°Gy ^28^Si (240 MeV/n), or 3°Gy of ^137^Cs gamma-rays at the NASA Space Research Laboratory (NSRL) building. While the shipping and irradiation conditions were the same between the cohorts, urine was collected from the second cohort 24 h after sham-irradiation or irradiation. Urine collection involved firmly holding the mouse over parafilm. In some cases, it was necessary to gently massage the mouse’s abdomen. Restraint time was generally, but not always, less than a minute. Urine was not collected from the first cohort. The week following irradiation, the mice were shipped to Colorado State University. Three months later, the mice were weighed and tested for contextual (hippocampus-dependent) and cued (amygdala-dependent and hippocampus-independent) fear conditioning. Potentially due to the additional stressor of the urine collection in the second cohort, there was a significant cohort effect and the mean and variance of a majority of phenotypes differed in the two cohorts of animals. Therefore, we analyzed the two cohorts separately and subsequently compared and contrasted the resulting findings.

### Fear Conditioning

The mice were tested for fear conditioning using Med Associates NIR Video and automated analysis (Med Associates, St. Albans, VT, United States) utilizing Med Associates Video Freeze automated scoring system. Pavlovian fear conditioning is a versatile and well-understood method of assessing associative learning and memory. In this task, mice learn to associate a conditioned stimulus (CS, e.g., a tone) with an unconditioned stimulus (US, e.g., a foot shock). CS–US pairings are preceded by a short habituation period, from which a baseline measure of locomotor activity is measured. On day 1, training, the mice were placed inside a white LED lit (100 lux) fear conditioning chamber (Context A). Context A consists of a metal grid floor with gray and white walls. There was a 90-s baseline followed by five CS–US pairings. During acquisition, the 30-s tone (CS) (80 db, 2,800 Hz) co-terminated with 2-s foot shocks (0.7 mA) (US). The inter-tone interval (ITI) was 90 s. Motion during shock (arbitrary units from proprietary index) was measured to explore potential treatment-induced differences in response to the aversive stimulus. Percent time freezing during each subsequent ITI and tone presentation was measured to assess acquisition of the fear response. On day 2, mice were exposed first to Context A for 300 s and to a new environment, Context B, 4 h later. Context B consists of a smooth white plastic floor, with a “tented” black plastic ceiling and scented with a 10% isopropanol solution. There was a 90-s baseline and a 180-s tone. Freezing was defined as the absence of motion with the exception of respiration. The freezing response is a widely used indicator of a conditioned fear response (Anagnostaras et al., 2001, 2010). Motion during shock (arbitrary units from proprietary index) was measured to evaluate potential treatment-induced differences in response to the aversive stimulus. Percent time freezing during each subsequent ITI and tone presentation was measured to assess acquisition of the fear response. On day 2, mice were first exposed to the training environment for 300 s and motion and freezing levels were analyzed. Subsequently, the mice were exposed to a new environment, consisting of a smooth white plastic floor, with a “tented” black plastic ceiling and scented with a 10% isopropanol solution. There was a 90-s baseline and a 180-s tone, i.e., a tone (CS), with a foot shock (US), and thereby come to fear the CS. Trained mice display this conditioned fear by ceasing all movement except for respiration in an attitude called “freezing.” Training takes place in a light and sound attenuated chamber (termed the “conditioning chamber”) equipped with a video camera. The type of training determines the brain regions involved in the learning and memory processes. The mice were tested for learning, memory, and extinction of hippocampus-dependent contextual fear conditioning using Med Associates NIR Video and automated analysis (Med Associates, St. Albans, VT, United States) utilizing Med Associates Video Freeze automated scoring system. Freezing was defined as the absence of motion with the exception of respiration. The freezing response is a widely used indicator of a conditioned fear response. On day 1 (training), each mouse was placed inside the enclosure with a house light (100 lux) on during the training trial. The test involved a 60-s baseline period in which the activity was recorded, followed by four tone–shock pairings. The tones were 80 dB at 2,800 Hz for 30 s, co-terminating with 2 s shocks (0.7 mA). Activity in the novel environment was defined as movement during the 60-s baseline period. The ITI was 90 s. Motion during shock (arbitrary units from proprietary index) was measured to evaluate potential treatment-induced differences in response to the aversive stimulus. Percent time freezing during each subsequent ITI and tone presentation was measured to assess acquisition of the fear response. On days 2 (day 1 of extinction) to 9 (day 8 of extinction), mice were first exposed to the training environment for 300 s and motion and freezing levels were analyzed. Subsequently, the mice were exposed to a new environment, consisting of a smooth white plastic floor, with a “tented” black plastic ceiling and scented with a 10% isopropanol solution. There was a 90-s baseline and a 180-s tone.

### Genotyping

DNA was prepared from tail snips (∼2 mm) collected at weaning. Each mouse was genotyped for 77.8 K SNP markers by GeneSeek (Lincoln, NE). We identified ∼40 K markers with sufficient genetic diversity (minor allele frequency above 5%). The mapping resolution was in the 200 kb to 2 Mb range for this HS mapping population.

### Multivariate Distance-Based QTL Detection

For the QTL detection, we used a modified version of multivariate distance matrix regression (MDMR), which relates *P* variables to *M* factors collected on *N* individuals, where *P* >> *N*. The most important feature of this procedure is the capacity to evaluate individual markers as well as groups of markers. In the present study, this feature was utilized to select groups of markers that collectively display the strongest association with the phenotype. MDMR analysis involves computing the distance between all pairs of individuals with respect to *P* variables of interest and constructing an *N* × *N* matrix whose elements reflect these distances. Permutation tests are used to test hypotheses and derive *p*-values that consider whether or not the *M* genetic factors (loci) can explain variation in the observed distances between and among the *N* individuals as reflected in the matrix of phenotype differences.

**FIGURE 1:**
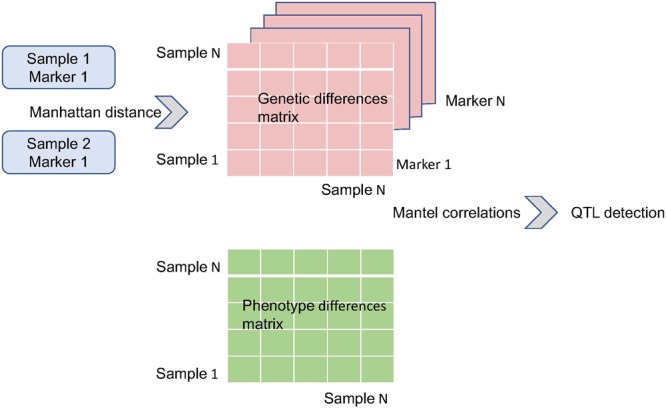
Outline of QTL detection utilizing Mantel correlations. Pairwise distances are computed between individuals/samples based on genotype at specific marker(s). This results in square matrix of genetic similarities between individuals. Multiple genetic markers can be included in the analysis by including several markers and applying the Manhattan distance to the resulting vector. A similar size matrix is computed utilizing pairwise distances in terms of phenotype. These genotype and phenotype matrices are correlated utilizing the Mantel procedure.

An essential step in the MDMR procedure involves selecting appropriate distance measures for each data modality – SNPs for the genotype and composite measures for the behavioral and cognitive data. For both the genotype data and phenotype data, we utilized the Manhattan distance, assigning equal weight to each genotype and phenotype. The essential steps of this approach are outlined in **Figure 1**.

For the purpose of finding optimal groups of genetic markers, we further combined the MDMR procedure with a pattern detection algorithm known as forward selection. Starting with the most significant genetic marker, we included additional markers to the analysis, selecting them in decreasing order of significance as quantified by the Mantel correlation with the phenotype. For each additional marker, the genotype distance matrix was recomputed and correlated once more with the phenotype distance matrix. The typical outcome of this procedure consists of initial increases in the cumulative/group correlation, followed by a peak reaching a plateau, after which including additional markers starts to decrease the correlation (**Figure 3A**).

While the forward selection procedure is often very powerful, it has the potential drawback of overfitting the data. To guard against this possibility, we employed two procedures. First, as outlined above, for each marker we computed the individual correlation (with the phenotype), as well as the associated *p-*value which was obtained by a resampling procedure in the Mantel test. Only markers individually significant at a *p*-value < 0.01 were included. Second, we randomized the data by shuffling the sample labels in the phenotype data and repeated the whole procedure, in effect producing an empirical distribution of results that can be obtained by pure chance. The real cumulative correlation value was combined with the empirical distribution of permutation-derived correlations and *Z-*scores were produced. This procedure facilitated selecting the number of markers that cumulatively produce robust and significant results.

### Statistical Analyses

Statistical analyses were performed in the R programming and analysis framework (R Foundation for and Statistical Computing, 2018).

## Results

### Summary and Interpretation of Behavioral and Cognitive Measurements

An initial examination of the behavioral data identified numerous phenotypes that displayed high correlations (Pearson *r* > 0.8 – see **Supplementary Table 1**). Most of the strong correlations were detected among related phenotypes. For example, correlations between the several “context (contextual fear memory)”-related phenotypes are in the range of 0.5–0.9. Similarly, high correlations were found when “cued” (cued fear memory) and “train” (learning day) phenotypes were compared within the same category. In contrast, we only detected small to moderate correlations (<0.6) among phenotypes that capture distinct behavioral and cognitive measures (e.g., between “cued” and “context” phenotypes). In most cases, we detected very low correlations, as in between “shock” (learning) and “context” phenotypes. These observations justified the grouping of related phenotypes into six main categories: *context_pctfrze* (*percent freezing during the contextual fear memory test*), *context_avgmot* (*average motion during the contextual fear memory test*), *cued_pctfrze* (*percent freezing during the cued fear memory test*), *train_pctfrze* (*percent freezing during acquisition*/*learning of fear memory on the training day*), *shock_avgmot* (*average motion during the shocks*), and *train_avgmo* (*average motion during the training*/*learning session*). To perform subsequent analyses and in particular multi-locus QTL mapping, related individual behavioral and cognitive measures were combined in a vector and, for each vector, we constructed pairwise distance matrices between all individuals.

### Cohort Effects in the Behavioral and Cognitive Measures

**FIGURE 2:**
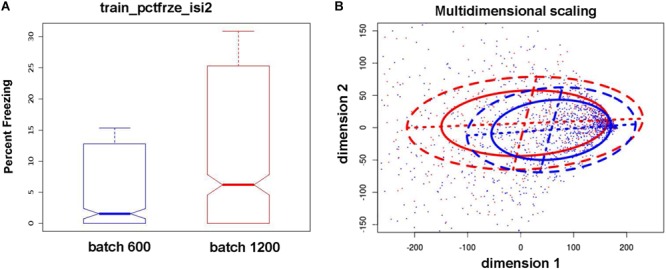
Differences in means and variance of phenotypes between the two batches/cohorts. These results illustrate the strong effects of the urine collection procedure on subsequent behavioral/cognitive measures. Blue: 600 cohort, red: 1,200 cohort. **(A)** Example boxplots for train_pctfrze_isi2 phenotype. The cognitive measure illustrated is the percent freezing during the second inter-stimulus interval, a measure of test learning. The mean percent freezing and variability in this measure was much larger in the cohort of 1,200 than the cohort of 600 mice. **(B)** Pairwise distances between samples are computed utilizing all train_pctfrze phenotypes. The resulting distances are projected on two dimensions and ellipses containing 50/75% of the data are drawn (solid and dashed lines, respectively). This illustrates the presence of different data distributions in both location and spread. Due to these differences our analysis was performed separately for each cohort.

Due to the large number of animals utilized in this study (∼1,800), the shipment to BNL and radiation and sham-irradiation there, and return shipment from BNL to CSU, behavioral and cognitive testing was performed in two cohorts, of ∼600 and ∼1,200 animals, respectively. For the second, but not first, cohort, there was urine collection 24 h after radiation or sham-irradiation. Due to the fact that the urine collection procedure and associated handling has the potential to serve as an additional environmental challenge and alter subsequent behavioral performance, we tested for the presence of differences in behavioral and cognitive measures between the two cohorts. For nearly all behavioral and cognitive measures we found significant differences in mean and variance [see **Figure 2** for an example involving the percent freezing during training in the fear conditioning test (*train_pctfrze* phenotype)]. This illustrates the sensitivity of the behavioral and cognitive measures assessed to alterations in the environment. Based on this result, subsequent analyses were performed independently for the two cohorts although we compared and contrasted quantitatively and qualitatively the findings from the two cohorts.

### Detection of Genomic Locations and Marker Sets Associated With Behavioral and Cognitive Measures

Behavioral and cognitive measures generally fall into the category of complex traits, which implies that they do not follow simple Mendelian models of inheritance and their genetic control is dispersed throughout the genome (Baud and Flint, 2017). Our analysis approach was selected based on this observation. Both phenotypes and genotypes were represented as multidimensional vectors; sample pairwise differences were computed between these vectors and correlated utilizing the Mantel procedure (Mantel, 1967); this general procedure has been previously utilized in both genetic (Wessel and Schork, 2006) and gene expression analyses (Shannon et al., 2002). We proceeded in three steps. First, we applied the procedure independently to each marker–phenotype pair and derived Mantel statistics (correlations) and associated *p*-values. Second, we utilized the forward selection machine learning technique and we combined the most significant markers for each phenotype and derive cumulative Mantel statistics. Third, we utilized randomization procedures to evaluate the robustness of this approach.

### Performance of the Distance-Based Multi-locus Genetic Association Procedure

**FIGURE 3:**
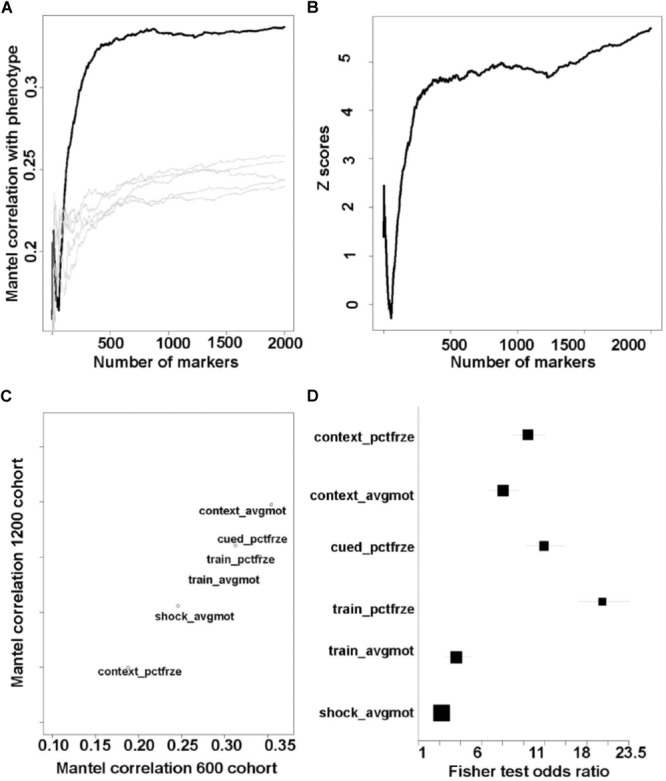
Significant markers sets are robustly detected in the two cohorts. These results illustrate that our QTL set detection procedure returns consistent results even under highly heterogeneous testing conditions. **(A)** Black line: QTL detection procedure applied to the context_avgmo phenotype. As more markers are included in the analysis the Mantel correlation reaches a plateau. When the same analysis is run on randomized order samples, the cumulative correlation does not reach significant values (gray lines). **(B)** *Z*-scores computed on the basis of the data in **A** (black line compared with gray lines). As more markers are included in the analysis, the *Z*-scores increase above 3, signifying that correlation values in **A** are unlikely to be achieved by chance alone. **(C)** Top genotype–phenotype values for the two cohorts are largely the same. **(D)** Overlap between significant markers detected independently in the two cohorts. For all phenotypes the size of overlap is significantly higher than what can be achieved by chance alone (confidence intervals for odds ratios >> 1). *P-*values in order from top to bottom: 8 × 10^-250^, 7 × 10^-159^, 5 × 10^-255^, ∼0, 1.06 × 10^-24^, 3 × 10^-18^.

As outlined above, our procedure starts from the most significant marker and expands the group of markers until either a peak is achieved or the individual marker significance *p*-value falls under 0.01. An example of the performance of this procedure is outlined in **Figure 3A** for the average movement during the contextual fear memory test (*context_avgmo*) phenotype. In this case, the performance (magnitude of the Mantel correlation) reached a plateau after 500 markers (thick black line in **Figure 3A**). We next repeated this procedure on randomized data (order of the samples in the phenotype datasets was scrambled). Examples of the resulting curves are illustrated in **Figure 3A** (thin gray lines). This procedure illustrates that peak performance in the original data (not randomized) was unlikely to be achieved by chance alone. We further quantified these results by constructing *Z*-statistics from the randomized and un-randomized data (**Figure 3B**); this revealed that as the number of markers grows beyond 2–300, *Z*-scores increase >3 (*p* < 0.0014) and eventually achieve levels >5 (*p* < 00001), is highly unlikely due to chance. Qualitatively similar results were obtained for all phenotypes (data not shown). We conclude that our procedure robustly detects groups of genetic markers that achieve a high correlation with the behavioral phenotypes. Importantly, both overall performance (correlation) and robustness are achieved when including larger number of genetic markers. Uncertainty remains about the influence of single markers, even when examining the marker achieving the greatest genome wide significance; this manifests in the data by sharp jumps in the significance and *Z*-score curves (**Figures 3A,B**).

### Detection and Comparison of Peak Genotype–Phenotype Cumulative Correlations

We detected sets of markers that cumulatively raised to as high as Mantel correlation of 0.35 (*p* < 0.001 based on 1,000 permutation tests) (*context_avgmo* phenotype – see **Figure 3A**). The average motion during the contextual fear memory test (*context_avgmo*) phenotype had the highest correlation with the genotype in both cohorts, while the percent freezing during the contextual fear memory test (*context_pctfrze*) had the lowest correlation (**Figure 3C**); the rankings of “genetic correlation” for the six composite phenotypes were identical in the 600 and 1,200 cohorts, as can be seen in **Figure 3C**. However, there was a significant shift downward in the magnitude of correlations between the 600 and 1,200 groups, as indicated by the departure from the diagonal of the linear relationships in **Figure 3C**. This set of results illustrates that: (1) all behavioral and cognitive measures had a significant genetic component, albeit dispersed among numerous genomic locations; (2) changes in environmental conditions between the two cohorts resulted in differences in genetic components of behavioral and cognitive measures, as quantified by the Mantel correlation; and (3) the ranking of the strength of the genetic control over behavioral and cognitive performance was preserved across the two cohorts.

### Environment Effects on the Genotype*–*Phenotype Correlations

Given the fact that the different testing environment in the two cohorts was associated with differences in the strength of genetic control over behavioral and cognitive performance, we inquired whether the identity of the significant genomic locations was altered as well. We utilized the Fisher exact test to quantify the level of overlap between sets of significant markers independently detected in each cohort. We found that for most behavioral and cognitive measures, the genomic locations that showed association with behavioral and cognitive performance shifted, with the average motion during training (*train_avgmo*) measure being the most affected (**Figure 3D**). We conclude that a change in the environmental conditions, which in our case was a recent intrusive urine collection, changed the identity of the genomic locations showing an association with certain behavioral and cognitive measures.

### Radiation Effects on the Genotype–Phenotype Correlations

**FIGURE 4:**
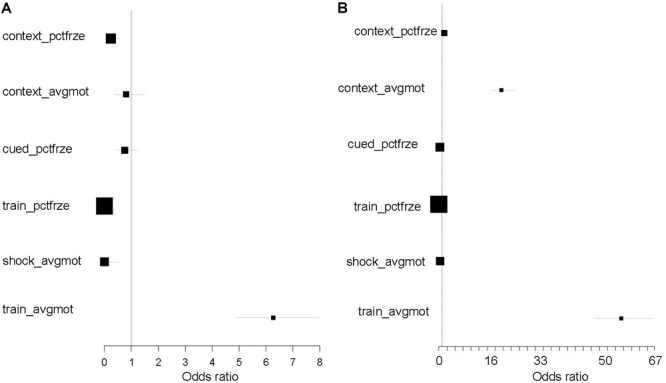
Exposure to ionizing radiation results in large shifts in the patterns of genetic influence over cognitive performance. Odds ratios below 1 signify that there is no overlap between the markers detected under sham versus ionizing radiation conditions. This lack of concordance in the identity of significant markers is detected in both the 600 cohort **(A)** and 1,200 cohort **(B)**. Only two phenotypes show concordance between sham and ionizing radiation: train_avgmo (both cohorts) and context_avgmo (1,200 cohort). Black filled squares represent confidence intervals of the Fisher exact test, with values < 1 showing lack of significant overlap.

Given the fact that shifts in environmental setting (urine testing) induced changes in phenotype–genotype correlations, we inquired whether radiation exposure has similar effects. This hypothesis was evaluated independently in the two cohorts. We separated the two main cohorts between the sham versus irradiated animals (combining all radiation types together). We then performed genetic scans independently in the irradiated and sham-irradiated animals. Comparing the identity of the sets of genetic markers detected revealed large shifts in their composition (**Figure 4**). Lack of overlap was quantified by the magnitude of the Fisher test odds ratio, with values below one being no different from chance. For nearly all behavioral and cognitive measures, the identity of the significant markers changed dramatically to the point that there was no overlap beyond what can be achieved by chance. It is important to note that the one phenotype that showed concordance between sham-irradiated and irradiated animals, average motion during training (*train_avgmo*) was the same in the two cohorts. We conclude that radiation exposure has the potential to alter phenotype–genotype correlations.

### Annotation of Genes Sets Associated With Selected Loci

We next inquired whether biological pathways or other functional groups are enriched in genes within the significant genomic locations. For each behavioral phenotype, we intersected the significant markers detected in each cohort. Next, we collected genes within 10^6^ base pairs of any of these common markers. We recognize that these gene sets might include some genes without direct association with the phenotype. However, since genes in chromosomal physical proximity often participate in common biological functions, this wide inclusion criteria can help facilitate the detection of relevant functional groups. Gene sets were evaluated for enrichment in gene members in Gene Ontology or KEGG pathways. We detected significant enrichment for a majority of phenotype/irradiation type combinations (**Supplementary Table 2**). For example, the average motion during training (*train_avgmo*) phenotype following sham-irradiation showed enrichment in “response to pheromone” and “response to chemical stimulus” GO functional categories. For the percent freezing during the contextual fear memory test (*context_pctfrze*) with Gamma radiation, we detected “regulation of proteolysis” and “negative regulation of endopeptidase activity.”

The phenotype-irradiation combinations with the most abundant annotations were context_pctfrze under ^56^Fe irradiation and train_avgmo under ^28^Si irradiation (**Supplementary Table 2**). These two phenotypes also displayed the strongest genetic basis in both batches (**Figure 3C**). Importantly, ^56^Fe and ^28^Si are two radiation types uniquely encountered in space flight. Given these findings, an in-depth examination of the specific GO and KEGG pathways uncovered by the genetic analysis is warranted.

Among the annotations associated with train_avgmo under ^28^Si, we have the PPAR_SIGNALING_PATHWAY (*p* < 2.2 × 10^-9^). This pathway is associated with regulation of peroxisome proliferation and adipocyte differentiation. However, emerging evidence suggests that within the adult mouse brain PPAR is highly expressed primarily not only in the hypothalamus, but also in the neocortex, the olfactory bulb, the organ of the vasculosum of the lamina terminalis (VOLT), and the subfornical organ (Liu et al., 2015). This study also found that within the hypothalamus, suprachiasmatic nucleus displays moderate levels of PPAR which is upregulated by fasting. Neurogenetic imaging has also revealed that genetic variability in PPAR is related to cerebral connectivity in preterm infants (Krishnan et al., 2017). Taken together, these findings provide indications of neurobehavioral roles of the PPAR pathway.

Among the annotation categories associated with train_avgmo under ^28^Si, we also find KEGG_MM_FATTY_ACID_METABOLISM (*p* < 5.7 × 10^-8^). There is extensive literature on the effect of ionizing radiation on fatty acid metabolism (Alfaia et al., 2007; Chukwuemeka et al., 2012; Mims et al., 2015; Rae et al., 2015). For KEGG_MM_RETINOL_METABOLISM (*p* < 1.6 × 10^-6^), there is evidence that retinoids reduce cellular apoptosis following irradiation (Vorotnikova et al., 2004); other studies have also found involvement of retinol in molecular or cellular responses to irradiation (Ross and Kempner, 1993).

Among the annotation categories associated with context_pctfrze under ^56^Fe irradiation, we find GO_MF_MM_ATPASE_ACTIVITY (*p* < 9.7 × 10^-7^). The mitochondrial activity of ATPASE has been shown to be affected by radiation in female mice (Kandil et al., 2018). Additionally, there have been early studies indicating changes in mouse serum esterase concentrations following ionizing radiation (Hunter et al., 1968); we also find several esterase-related annotations associated with context_pctfrze under ^56^Fe irradiation (**Supplementary Table 2**).

## Discussion

Our main finding is that phenotype/genotype correlations are plastic and can shift depending on the external environment, and in particular following ionizing radiation. Recent studies have revealed that a significant proportion of QTLs reported in the literature fail to replicate in subsequent studies (Schielzeth et al., 2018). Given these findings, we leveraged the presence of two distinct cohorts and exposure to different types of radiation to not only detect significant QTL groups, but more importantly to evaluate their reproducibility across cohorts and radiation types. Our work therefore contributes to the understanding of causes of the varying levels of reproducibility affecting QTL studies.

This observation has important implications regarding potential effects of space radiation on behavioral and cognitive performance. Our results suggest that complex behaviors and their genetic basis have to be studied under realistic conditions, which in our case means irradiated animals. Behavioral QTLs from non-irradiated animals will have limited predictive power once the animals are subjected to irradiation.

We find that alterations in environmental conditions and environmental challenges other than irradiation also have the potential to alter behavioral and cognitive performance and their genetic basis, an observation consistent with previous findings (Champagne and Mashoodh, 2009; Beery and Kaufer, 2015). Stressful events (such as intrusive urine collection) have a strong effect on the actual behavioral and cognitive performance (Kurien et al., 2004). It is important to note that there is a qualitative distinction between the effects of stress versus the effects of ionizing radiation. Stressful events lead to actual shift in behavioral and cognitive performance, but do not completely alter the genotype/phenotype correlations (odds ratios ≫1 in **Figure 3D**). In contrast, ionizing radiation does not directly alter behavioral and cognitive performance but has profound effects on the genotype/phenotype correlations (**Figure 4**). It is striking that in the larger cohort of 1,200 mice in which there was urine collection 24 h following radiation exposure, the mean percent freezing during training, a measure of learning, as well as the variability in this measure in individual mice was much higher than that in smaller cohort of 600 mice in which there was no urine collection (**Figure 2A**). Striking cohort effects were also seen in **Figure 4**. There was more overlap between the genetic effect on activity during the contextual fear memory test. This was detected the day following training and between irradiated and sham-irradiated mice in the cohort of 1,200 mice. As noted above, in the 1,200 mice cohort there was urine collection 24 h following radiation exposure. In contrast, in the cohort of 600 mice cohort there was no urine collection. In other words, there appear long-term effects of urine collection that diminishes the ability to detect genetic effects of irradiation on behavioral performance in a hippocampus-dependent fear memory test. No such difference was seen for hippocampus-independent cued fear memory. These data indicate that the hippocampus might be particularly sensitive to long-term effects of environmental stressors that might affect radiation effects. However, the cohort effects were also seen when activity during fear conditioning training was compared. The odds ratio for activity during the training session was much higher in the cohort of 1,200 than in the cohort of 600 mice. These data suggest that the urine collection causes a dramatic increase in overlap between the genetic effects on activity during the session in which the mice learned this task. As astronauts during space missions encounter environmental challenges in addition to radiation exposures, increased efforts are warranted to determine how environmental challenges other than radiation might modulate long-term effects of space radiation on the brain.

Numerous studies have encountered and commented on the varying levels of reproducibility of genetic association studies (Shen et al., 2005; Li and Meyre, 2013; Bian et al., 2014; Schielzeth et al., 2018). Benefiting from moderate sample sizes over each batch/radiation type combination, our study addresses the issue of reproducibility in a systematic manner. It is important to note that since we do not focus on individual markers or genes but on sets that collectively achieve high correlation with the phenotype, this confers an increased level of robustness to the findings.

Within behavioral genetics there is wide recognition that Mendelian mutations leading to large shifts in behavioral and cognitive performance are extremely rare (Flint, 2003). Given that many behavioral and cognitive measures are still clearly under genetic control, this observation points to the possibility that combinations of genetic loci of small-to-moderate effects collectively control behavioral and cognitive performance. The multi-locus analysis methodology employed here is well suited to perform analyses under these conditions. We note that we utilized a simple identity by state (IBS) distance matrix (Wessel and Schork, 2006); alternatives include weighing each marker by its allele frequency or by functional role.

Our annotation approach closely follows (Avramopoulos et al., 2006) and is based on formulation of hypotheses that do not focus on single genes but instead consider groups of genes grouped either based on functional roles (pathways and ontologies) or based on location (vicinity to a marker of moderate significance). The critical assumption of this approach is that the two gene groups are constructed independently, an assumption satisfied in our study. As noted in Avramopoulos et al. (2006), when the relevant functional unit is a group of genes (a pathway), one can expect that the genetic markers falling within these genes will have increased individual significance scores, even though these will be within the stochastic noise range and far from achieving the genome-wide significance of top results in a GWAS study. However, *when considered as a group*, their collective significance will clearly point to the implication of the relevant pathway.

We examined closely some of the pathways associated with the two behavioral phenotypes with the strongest genetic component, context_pctfrze under ^56^Fe irradiation and train_avgmo under ^28^Si irradiation. There are several conclusions that we draw from this analysis. First, at the level of annotations and pathways, there is no overlap in the categories detected for different radiation types and behavioral and cognitive phenotypes. This finding suggests that distinct radiation types lead to the “engagement” of different biological mechanisms, pathways, and ontologies. This observation is concordant with previous experimental work showing that irradiation parameters induce different types of DNA damage (Prise et al., 1994), and also with modeling work aligning distinct irradiation features with distinct patterns of DNA damage.

At the conceptual level, our findings suggest the following model of interaction between irradiation, genetics, and behavioral and cognitive patterns. Ionizing radiation engenders effects on behavioral and cognitive performance, and internal compensatory mechanisms mitigate these effects. However, genetics plays a part as to which compensatory mechanisms are engaged in each individual animal. Individual genetic variability interacts with variation in the irradiation type, resulting in heterogeneity in the identity of individual genetic loci and of the functional pathways affected.

## Ethics Statement

The experiments were approved by the IACUC committees from BNL and CSU.

## Author Contributions

OI, JB, MW, and JR designed the experiments and wrote the manuscript. SB, RO, MD, BS, ME, TM, CF, and EE acquired and analyzed the data and wrote the manuscript.

## Conflict of Interest Statement

The authors declare that the research was conducted in the absence of any commercial or financial relationships that could be construed as a potential conflict of interest.
